# SARS-CoV-2 seroprevalence in the urban population of Qatar: An analysis of antibody testing on a sample of 112,941 individuals

**DOI:** 10.1016/j.isci.2021.102646

**Published:** 2021-05-24

**Authors:** Peter V. Coyle, Hiam Chemaitelly, Mohamed Ali Ben Hadj Kacem, Naema Hassan Abdulla Al Molawi, Reham Awni El Kahlout, Imtiaz Gilliani, Nourah Younes, Ghada Ali A.A. Al Anssari, Zaina Al Kanaani, Abdullatif Al Khal, Einas Al Kuwari, Adeel A. Butt, Andrew Jeremijenko, Anvar Hassan Kaleeckal, Ali Nizar Latif, Riyazuddin Mohammad Shaik, Hanan F. Abdul Rahim, Gheyath K. Nasrallah, Hadi M. Yassine, Mohamed Ghaith Al Kuwari, Hamad Eid Al Romaihi, Mohamed H. Al-Thani, Roberto Bertollini, Laith J. Abu-Raddad

**Affiliations:** 1 Hamad Medical Corporation, Doha, P.O. Box 3050, Qatar; 2 Biomedical Research Center, Qatar University, Doha, P.O. Box 2713, Qatar; 3 Wellcome-Wolfson Institute for Experimental Medicine, Queens University, Belfast, BT7 1NN United Kingdom; 4 Infectious Disease Epidemiology Group, Weill Cornell Medicine-Qatar, Cornell University, Qatar Foundation - Education City, P.O. Box 24144, Doha, Qatar; 5 World Health Organization Collaborating Centre for Disease Epidemiology Analytics on HIV/AIDS, Sexually Transmitted Infections, and Viral Hepatitis, Weill Cornell Medicine–Qatar, Cornell University, Qatar Foundation – Education City, Doha, P.O. Box 24144, Qatar; 6 College of Health Sciences, QU Health, Qatar University, Doha, P.O. Box 2713, Qatar; 7 Department of Biomedical Science, College of Health Sciences, Member of QU Health, Qatar University, Doha, P.O. Box 2713, Qatar; 8 Primary Health Care Corporation, Doha, P.O. Box 26555, Qatar; 9 Ministry of Public Health, Doha, P.O. Box 42, Qatar; 10 Department of Population Health Sciences, Weill Cornell Medicine, Cornell University, New York, NY 10065, USA

**Keywords:** Health informatics, Public health, Infection control in health technology, Immunology

## Abstract

The study objective was to the assess level of detectable severe acute respiratory syndrome coronavirus 2 (SARS-CoV-2) antibodies in the urban population of Qatar. Antibody testing was performed on residual blood specimens for 112,941 individuals (∼10% of Qatar's urban population) attending for routine/other clinical care between May 12 and September 9, 2020. Seropositivity was 13.3% (95% confidence interval [CI] = 13.1–13.6%) and was independently associated with sex, age, nationality, clinical care encounter type, and testing date. Median optical density (antibody titer) among antibody-positive persons was 27.0 (range = 1.0–150.0), with higher values associated with age, nationality, clinical care encounter type, and testing date. Seropositivity by nationality was positively correlated with the likelihood of having higher antibody titers (Pearson correlation coefficient = 0.85; 95% CI = 0.47–0.96). Less than two in every 10 individuals in Qatar's urban population had detectable antibodies against SARS-CoV-2, suggesting this population is still far from herd immunity and at risk of subsequent infection waves. Higher antibody titer appears to be a biomarker of repeated exposures to the infection.

## Highlights

• Less than 20% of Qatar's urban population had detectable antibodies for SARS-CoV-2
• Qatar's urban population is still at risk of future epidemic waves
• Antibody titers varied by age, nationality, and over time
• Higher antibody titers appear to be a biomarker of re-exposures to the infection

## Graphical abstract

**Figure:**
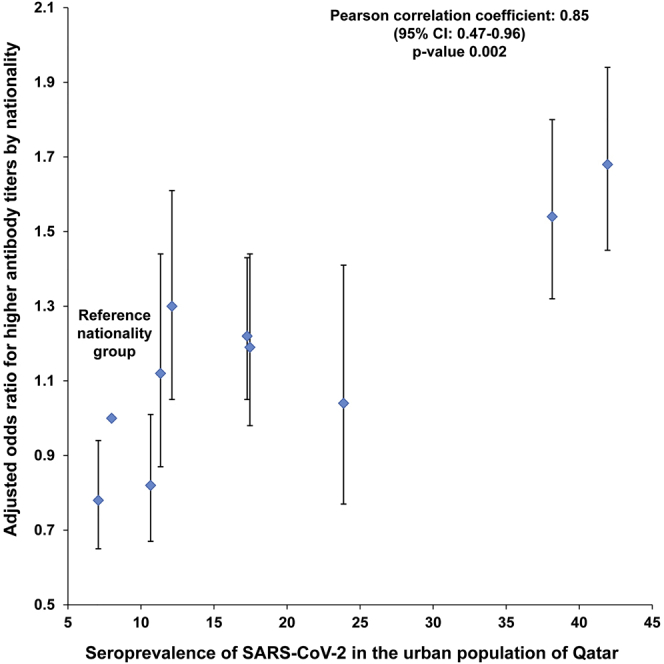

## Introduction

With the breakthrough development of highly efficacious vaccines against the severe acute respiratory syndrome coronavirus 2 (SARS-CoV-2) (Polack et al., 2020; Jackson et al., 2020; Voysey et al., 2020), determining the population's cumulative infection exposure and current immunity level is critical to inform national vaccine roll-out strategies.

Qatar, located in the Arabian Peninsula, with a multinational population of 2.8 million people, nearly all living in the capital city, Doha, had a significant first wave of coronavirus disease 2019 (COVID-19) that peaked in late May 2020 (Planning and Statistics Authority- State of Qatar, 2020; Planning and Statistics Authority-State of Qatar, 2019). As of December 23, 2020, >60,000 infections per million population (number of infections divided by total population) had been laboratory confirmed (Hamad Medical Corporation, 2020b; Ministry of Public Health-State of Qatar, 2021). Qatar has a unique socio-demographic structure, in which single-unit and family households, including children, adults, and/or older adults, account for only 40% of the total population, with adults in this “urban population” often being part of the professional or service workforce (Planning and Statistics Authority- State of Qatar, 2020; Ministry of Interior-State of Qatar, 2020; Planning and Statistics Authority- State of Qatar, 2017). The remaining 60% of the population consists of craft and manual workers (CMWs) (Planning and Statistics Authority- State of Qatar, 2020; Ministry of Interior-State of Qatar, 2020; Planning and Statistics Authority- State of Qatar, 2017)—mostly single, young men working in development projects (Planning and Statistics Authority- State of Qatar, 2017) and typically living in large, shared accommodations (De Bel-Air, 2018).

Infection transmission in Qatar was first documented among CMWs on March 6, 2020 (Al Kuwari et al., 2020), who were subsequently most affected by this epidemic (Abu-Raddad et al., 2021a). A recently completed nationwide, population-based survey assessing “every” infection among the CMW population found that six out of every ten persons had detectable antibodies against SARS-CoV-2 (Al-Thani et al., 2021; Jeremijenko et al., 2021), suggesting that this population is at or near herd immunity for the variants circulating at this time (Jeremijenko et al., 2021; Anderson et al., 2020; Britton et al., 2020). In the present study, the first objective was to assess the level of infection exposure among the rest of the population of Qatar, that of the “urban population” of this country. The urban population was defined as the complement of the CMW population, that is, the population that lives in single-unit or family households (not shared accommodations) and is part of the professional or service workforce. This part of the population is highly diverse and includes over 150 nationalities in addition to Qataris. The second objective was to identify predictors for infection and for having higher antibody titers.

## Results

In all, 112,941 individuals were tested for SARS-CoV-2 antibodies, representing ∼10% of the urban population of Qatar (Planning and Statistics Authority- State of Qatar, 2020) (Table 1). Of these, 51.6% were men. Two-thirds (66%) of tested persons were 20–49 years of age. Qatari (25.8%) and Indian nationals (16.5%) were most heavily represented in the sample, reflecting their representation in the urban population (Ministry of Interior-State of Qatar, 2020; Priya Dsouza Communications, 2019; Planning and Statistics Authority-State of Qatar, 2019). Blood specimens were collected in the course of routine clinical care during home care visits (34.2%), outpatient visits (28.5%), inpatient hospital stays (21.0%), and emergency department visits (16.4%). Overall, the sample mirrored the urban population demographics (Table S1 of supplemental information (SI)) (Primary Health Care Corporation, 2020, Ministry of Interior-State of Qatar, 2020; Planning and Statistics Authority- State of Qatar, 2017; Planning and Statistics Authority-State of Qatar, 2019).

**Table 1:** Characteristics of tested individuals (112,941) and antibody positivity

Characteristics	Tested	Antibody positive		Univariable regression analysis		F Test	Multivariable regression analysis	
	N (%)	N (%^a^)	p value	OR^a^ (95% CI)	p value	p value^b^	AOR^a^ (95% CI)	p value^c^
Sex								

Women	54,707 (48.4)	4,387 (8.0)	<0.001	1.00			1.00	
Men	58,234 (51.6)	14,457 (18.3)		2.59 (2.44–2.73)	<0.001	<0.001	2.07 (1.95–2.21)	<0.001

Age (years)								
<10	3,384 (3.0)	243 (7.1)	<0.001	1.00			1.00	
10–19	5,557 (4.9)	407 (7.3)		1.04 (0.87–1.25)	0.633	<0.001	1.21 (1.00–1.46)	0.049
20–29	19,271 (17.1)	2,867 (15.0)		2.33 (2.03–2.69)	<0.001		2.04 (1.76–2.36)	<0.001
30–39	31,622 (28.0)	5,533 (16.8)		2.67 (2.33–3.06)	<0.001		2.21 (1.91–2.55)	<0.001
40–49	23,582 (20.9)	4,876 (18.1)		2.91 (2.53–3.34)	<0.001		2.47 (2.14–2.85)	<0.001
50–59	16,363 (14.5)	3,220 (17.9)		2.87 (2.50–3.31)	<0.001		2.46 (2.13–2.85)	<0.001
60–69	8,639 (7.6)	1,281 (15.0)		2.32 (2.00–2.69)	<0.001		2.12 (1.82–2.46)	<0.001
70–79	3,192 (2.8)	315 (10.5)		1.54 (1.29–1.84)	<0.001		1.73 (1.44–2.07)	<0.001
80+	1,331 (1.2)	102 (7.5)		1.06 (0.83–1.36)	0.621		1.42 (1.10–1.83)	0.007

Nationality								
All other nationalities^d^	24,799 (22.0)	2,203 (8.0)	<0.001	1.00		<0.001	1.00	
Bangladeshi	7,773 (6.9)	3,471 (41.9)		8.32 (7.66–9.04)	<0.001		5.05 (4.63–5.50)	<0.001
Nepalese	4,962 (4.4)	2,236 (38.2)		7.10 (6.43–7.85)	<0.001		4.26 (3.87–4.69)	<0.001
Pakistani	5,114 (4.5)	1,419 (23.9)		3.61 (3.23–4.04)	<0.001		3.45 (3.07–3.87)	<0.001
Indian	18,590 (16.5)	4,114 (17.5)		2.44 (2.24–2.65)	<0.001		1.95 (1.80–2.13)	<0.001
Sri Lankan	2,252 (2.0)	556 (17.3)		2.40 (2.06–2.81)	<0.001		1.85 (1.60–2.13)	<0.001
Filipino	7,085 (6.3)	1,100 (12.1)		1.59 (1.42–1.77)	<0.001		1.59 (1.41–1.78)	<0.001
Sudanese	3,954 (3.5)	466 (11.3)		1.47 (1.25–1.73)	<0.001		1.36 (1.15–1.61)	<0.001
Egyptian	9,329 (8.3)	1,150 (10.7)		1.38 (1.23–1.53)	<0.001		1.33 (1.19–1.48)	<0.001
Qatari	29,083 (25.8)	2,129 (7.1)		0.88 (0.80–0.97)	0.008		0.95 (0.86–1.04)	0.266

Clinical care encounter type								
Emergency	18,473 (16.4)	3,333 (14.2)	<0.001	1.00		<0.001	1.00	
Inpatient	23,720 (21.0)	6,308 (19.4)		1.45 (1.35–1.57)	<0.001		1.19 (1.10–1.28)	<0.001
Outpatient	32,146 (28.5)	5,264 (13.1)		0.91 (0.85–0.98)	0.011		0.87 (0.81–0.94)	0.001
Home care/follow-up consultations	38,602 (34.2)	3,939 (9.2)		0.61 (0.56–0.66)	<0.001		0.72 (0.66–0.78)	<0.001

Antibody test date								
Calendar date (a linear term)	112,941 (100.0)	18,844 (13.3)	<0.001	0.999 (0.998–1.000)	0.123	0.123	1.002 (1.001–1.003)	<0.001

AOR, adjusted odds ratio; CI, confidence interval; OR, odds ratio.

a Estimates are proportions of antibody-positive persons among those tested, weighted by sex, age, and nationality.

b Covariates with p value ≤0.2 in the univariable analysis were included in the multivariable analysis.

c Covariates with p value ≤0.05 in the multivariable analysis were considered strong predictors of anti-SARS-CoV-2 positivity after adjustment for sex, age, nationality, clinical care encounter type, and antibody test date.

d These include other nationalities residing in Qatar.

A total of 18,844 individuals had detectable SARS-CoV-2 antibodies—a weighted antibody positivity of 13.3% (95% confidence interval [CI]: 13.1–13.6%). Seropositivity was independently associated with sex, age, nationality, clinical care type, and calendar date of the antibody test in the multivariable regression analysis (Table 1). Men had two-fold higher odds of being seropositive (adjusted odds ratio [AOR] of 2.07; 95% CI: 1.95–2.21) than women. Similarly, the AOR was two-fold higher for adults aged 20–79 years than for children <10 years. Seropositivity varied by nationality. Compared to other nationalities, AOR was 5.05 (95% CI: 4.63–5.50) for Bangladeshis, 4.26 (95% CI: 3.87–4.69) for Nepalese, 3.45 (95% CI: 3.07–3.87) for Pakistanis, 1.95 (95% CI: 1.80–2.13) for Indians, 1.85 (95% CI: 1.60–2.13) for Sri Lankans, 1.59 (95% CI: 1.41–1.78) for Filipinos, 1.36 (95% CI: 1.15–1.61) for Sudanese, 1.33 (95% CI: 1.19–1.48) for Egyptians, and 0.95 (95% CI: 0.86–1.04) for Qataris. Compared to emergency department attendees, AOR was 0.87 (95% CI: 0.81–0.94) for outpatients and 0.72 (95% CI: 0.66–0.78) for patients with home care visits or follow-up consultations and 1.19 (95% CI: 1.10–1.28) for inpatients. There was evidence of increasing seropositivity over time (Table 1 and Table S2 of supplemental information) but at a slow rate. The AOR (per day) was only 1.002 (95% CI: 1.001–1.003; Table 1). Figure S1 of supplemental information shows seropositivity month by month, which was largely stable over the study duration.

**Figure 1:**
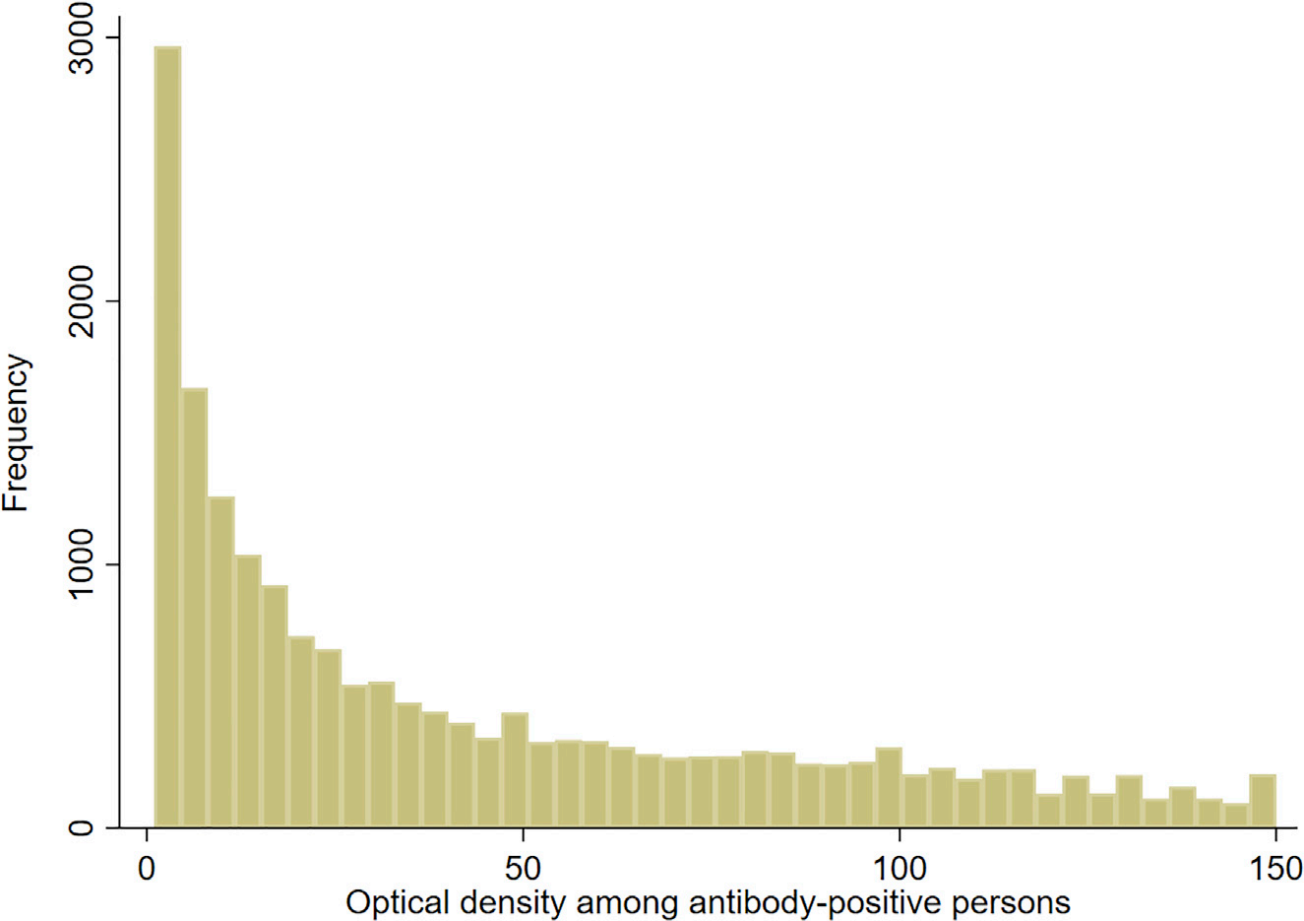
Distribution of antibody titers (optical density values) among the 18,844 antibody-positive individuals identified in this study

Figure 1 illustrates the distribution of antibody titers (optical density values) among the 18,844 antibody-positive persons. Optical density values ranged from 1.0 to 150.0 with a median of 27.0. Having higher antibody titers than the median was not associated with sex, but in the multivariable regression analysis, they were independently associated with age, nationality, clinical care type, and the calendar date of the antibody test (Table 2). Compared to those aged 20–29 years, the AOR was higher in children <10 years and adults aged 40–79 years. There were significant differences by nationality. AOR was 1.68 (95% CI: 1.45–1.94) for Bangladeshis, 1.54 (95% CI: 1.32–1.80) for Nepalese, 1.30 (95% CI: 1.05–1.61) for Filipinos, 1.22 (95% CI: 1.05–1.43) for Indians, 1.19 (95% CI: 0.98–1.44) for Pakistanis, 1.12 (95% CI: 0.87–1.44) for Sri Lankans, 1.04 (95% CI: 0.77–1.41) for Sudanese, 0.82 (95% CI: 0.67–1.01) for Egyptians, and 0.78 (95% CI: 0.65–0.94) for Qataris. Compared to emergency department attendees, inpatients had an AOR for higher antibody positivity of 0.38 (95% CI: 0.34–0.43), while no difference was found for outpatients or for patients with home care visits or follow-up consultations. Having higher antibody titers increased with time (Tables 2 and S3 of supplemental information), with an AOR (per day) of 1.011 (95% CI: 1.010–1.013; Table 2).

**Table 2:** Associations of antibody titers (optical densities) higher than the median value (≥27.0) among the 18,844 antibody-positive individuals

Characteristics	Tested	Higher antibody titers than the median		Univariable regression analysis		F Test	Multivariable regression analysis	
	N (%)	N (%^a^)	p value	OR^a^ (95% CI)	p value	p value^b^	AOR^a^ (95% CI)	p value^c^
Sex								

Women	4,387 (23.3)	2,175 (52.1)	0.702	1.00		0.702	–	–
Men	14,457 (76.7)	7,244 (51.6)		0.98 (0.88–1.09)	0.702		–	–

Age (years)								
20–29^d^	2,867 (15.2)	1,360 (47.6)	<0.001	1.00		<0.001	1.00	
<10	243 (1.3)	170 (70.0)		2.57 (1.92–3.45)	<0.001		2.85 (2.12–3.83)	<0.001
10–19	407 (2.2)	210 (54.0)		1.30 (1.02–1.65)	0.036		1.19 (0.90–1.56)	0.220
30–39	5,533 (29.4)	2,701 (48.1)		1.02 (0.92–1.13)	0.692		0.98 (0.88–1.09)	0.678
40–49	4,876 (25.9)	2,442 (50.1)		1.11 (1.00–1.22)	0.051		1.13 (1.02–1.26)	0.025
50–59	3,220 (17.1)	1,634 (50.8)		1.14 (1.02–1.27)	0.020		1.20 (1.07–1.35)	0.002
60–69	1,281 (6.8)	700 (54.2)		1.31 (1.14–1.50)	<0.001		1.46 (1.26–1.70)	<0.001
70–79	315 (1.7)	158 (49.7)		1.09 (0.86–1.38)	0.480		1.38 (1.07–1.77)	0.012
80+	102 (0.5)	44 (41.7)		0.79 (0.52–1.19)	0.254		1.27 (0.82–1.97)	0.281

Nationality								
All other nationalities^e^	2,203 (11.7)	1,064 (51.7)	<0.001	1.00		<0.001	1.00	
Bangladeshi	3,471 (18.4)	1,946 (56.2)		1.20 (1.04–1.38)	0.012		1.68 (1.45–1.94)	<0.001
Nepalese	2,236 (11.9)	1,099 (49.3)		0.91 (0.78–1.06)	0.210		1.54 (1.32–1.80)	<0.001
Filipino	1,100 (5.8)	507 (46.2)		0.80 (0.65–0.99)	0.038		1.30 (1.05–1.61)	0.017
Indian	4,114 (21.8)	2,027 (51.0)		0.97 (0.84–1.13)	0.699		1.22 (1.05–1.43)	0.011
Pakistani	1,419 (7.5)	731 (55.5)		1.16 (0.96–1.41)	0.124		1.19 (0.98–1.44)	0.076
Sri Lankan	556 (3.0)	242 (45.2)		0.77 (0.59–0.99)	0.044		1.12 (0.87–1.44)	0.392
Sudanese	466 (2.5)	264 (53.2)		1.06 (0.78–1.44)	0.712		1.04 (0.77–1.41)	0.789
Egyptian	1,150 (6.1)	535 (49.0)		0.90 (0.73–1.10)	0.283		0.82 (0.67–1.01)	0.062
Qatari	2,129 (11.3)	1,004 (51.8)		1.00 (0.84–1.21)	0.959		0.78 (0.65–0.94)	0.010

Clinical care encounter type								
Emergency	3,333 (17.7)	1,866 (58.5)	<0.001	1.00		<0.001	1.00	
Outpatient	5,264 (27.9)	3,189 (60.7)		1.10 (0.97–1.25)	0.136		1.06 (0.94–1.21)	0.334
Home care/follow-up consultations	3,939 (20.9)	2,195 (58.3)		0.99 (0.86–1.14)	0.915		1.00 (0.87–1.15)	0.987
Inpatient	6,308 (33.5)	2,169 (35.2)		0.39 (0.34–0.44)	<0.001		0.38 (0.34–0.43)	<0.001

Antibody test date								
Calendar date (a linear term)	18,844 (100.0)	9,419 (51.8)	<0.001	1.014 (1.012–1.016)	<0.001	<0.001	1.011 (1.010–1.013)	<0.001

AOR, adjusted odds ratio; CI, confidence interval; OR, odds ratio.

a Estimates are proportions of persons with antibody titers higher than the median among those antibody-positive, weighted by sex, age, and nationality.

b Covariates with p value ≤0.2 in the univariable analysis were included in the multivariable analysis.

c Covariates with p value ≤0.05 in the multivariable analysis were considered strong predictors of anti-SARS-CoV-2 positivity after adjustment for age, nationality, clinical care encounter type, and antibody test date.

d The 20–29 age group was chosen as the reference group (instead of the <10 age group) because of the larger sample size and for epidemiological relevance.

e These include other nationalities residing in Qatar.

**Figure 2:**
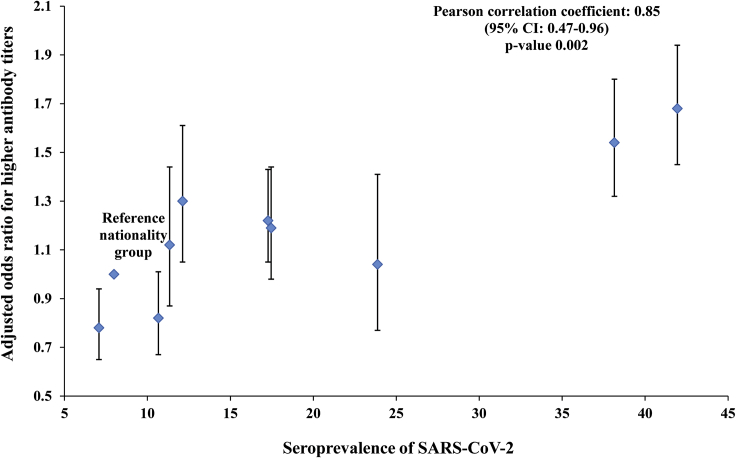
Adjusted odds ratios for higher antibody titers (optical densities higher than the median value of 27.0) for each nationality (Table 2) versus the corresponding SARS-CoV-2 seroprevalence for that nationality (Table 1). Error bars indicate 95% confidence intervals.

There was a strong correlation between the AOR for higher antibody titers in each nationality and the corresponding SARS-CoV-2 seroprevalence of that nationality (Figure 2). The Pearson correlation coefficient was 0.85 (95% CI: 0.47–0.96), possibly indicating that higher antibody titers correlate with repeated exposures to this coronavirus.

Of the 18,844 antibody-positive persons, 9,375 had a polymerase chain reaction (PCR)-confirmed diagnosis prior to the antibody-positive test—47.1% (95% CI: 46.1–48.2%) (Table 3). Meanwhile, 1,085 of the 18,844 antibody-positive persons had or progressed to a severe infection and 393 had or progressed to critical infection. Thus, the infection severity rate was 3.9% (95% CI: 3.7–4.2%), the infection criticality rate was 1.3% (95% CI: 1.1–1.4%), and the combined infection severity or criticality rate was 5.2% (95% CI: 4.9–5.5%). With exactly 100 COVID-19 deaths recorded among the antibody-positive persons, the infection fatality rate was 0.3% (95% CI: 0.2–0.3%).

**Table 3:** Key SARS-CoV-2 epidemiological measures assessed in the study.

Epidemiological measure	Sample (denominator)	Positive for outcome (numerator)	Estimate in percentage (95% CI)
Sample antibody positivity (seropositivity)	112,941	18,844	16.7 (16.5–16.9)
Weighted antibody positivity (seropositivity)	112,941	18,844	13.3 (13.1–13.6)^a^
Proportion with prior PCR-confirmed diagnosis^b^	18,844	9,375	47.1 (46.1–48.2)^a^
Infection severity rate^c^	18,844	1,085	3.9 (3.7–4.2)^a^
Infection criticality rate^d^	18,844	393	1.3 (1.1–1.4)^a^
Infection severity or criticality rate^e^	18,844	1,478	5.2 (4.9–5.5)^a^
Infection fatality rate^f^	18,844	100	0.3 (0.2–0.3)^a^

PCR, polymerase chain reaction; CI, confidence interval.

a Estimates weighted by sex, age, and nationality.

b Proportion of antibody-positive persons who had a prior SARS-CoV-2 PCR-confirmed diagnosis.

c Number of infections that were severe per World Health Organization criteria (World Health Organization, 2020) over the total number of antibody-positive persons.

d Number of infections that were critical per World Health Organization criteria (World Health Organization, 2020) over the total number of antibody-positive persons.

e Number of infections that were severe or critical per World Health Organization criteria (World Health Organization, 2020) over the total number of antibody-positive persons.

f Number of COVID-19 deaths per World Health Organization criteria (World Health Organization, 2020) over the total number of antibody-positive persons.

## Discussion

The above results indicate that <20% of the urban population of Qatar, which constitutes ∼40% of the total population and includes nearly all older adults, manifests evidence of prior infection. This seroprevalence is substantially less than that in the CMW part of the population, which was estimated recently in a nationwide survey at 55.3% (Al-Thani et al., 2021), with the leading risk factor being living in a large shared accommodation (Al-Thani et al., 2021; Abu-Raddad et al., 2021a; Jeremijenko et al., 2021; Al Kuwari et al., 2020).

This finding suggests that the lockdown and imposed social and physical distancing restrictions have been more successful in slowing transmission in the urban population compared to the CMW population. Building on the totality of evidence on the Qatar epidemic (Abu-Raddad et al., 2021a; Al Kuwari et al., 2020; Al-Thani et al., 2021; Ayoub et al., 2021; Jeremijenko et al., 2021), this appears to be due to the dwelling structure, in that the urban population lives mostly in single-unit or family households that each includes a small number of individuals. Meanwhile, the CMW population lives mostly in large shared accommodations that each includes a large number of individuals. While the lockdown forced individuals to stay more at place of residence, it is typical to have more social contacts every day in a large shared accommodation than in a single-unit or family household. This outcome highlights the role of the “boarding school” effect in respiratory infection transmission, seen often in the intense influenza outbreaks in regular and boarding schools (Jackson et al., 2013; Glatman-Freedman et al., 2012). This effect has been also seen in the large SARS-CoV-2 outbreaks in nursing homes in Europe and the United States (Arons et al., 2020; Burton et al., 2020; Ladhani et al., 2020).

With a seroprevalence of <20%, the urban population of Qatar remains far below the herd immunity threshold, estimated at 60–70% infection exposure (Anderson et al., 2020; Britton et al., 2020; Jeremijenko et al., 2021). Accordingly, there should exist potential for subsequent waves of infection in this part of the population, but no second wave materialized from May 2020 up to end of this year (Abu-Raddad et al., 2021a; Ayoub et al., 2021), that is, before the introduction and expansion of the B.1.1.7 and B.1.351 variants of concern (Abu-Raddad et al., 2021b). On the contrary, only a slow increase in seroprevalence has occurred since the peak of the first wave (Table S2 of supplemental information), reflecting the actual low incidence of infection in Qatar up to end of 2020 (Ayoub et al., 2021). The absence of a new wave, despite the lack of a lockdown and easing of many social distancing restrictions, may be explained by an “immunity shield” effect (Weitz et al., 2020) arising from the social mixing between the urban and CMW populations. With most CMWs being immune, the infection transmission chains had difficulty in sustaining themselves, as they were interrupted by the presence of immune persons who were not getting reinfected (Abu-Raddad et al., 2021b, 2021c; Abu-Raddad et al., 2020. ciaa1846. https://doi.org/10.1093/cid/ciaa1846). The effective implementation of “$R_{t}$ tuning,” an adjustment of restrictions by national policymakers based on the $R_{t}$ value, may have also contributed to preventing a new wave (Ayoub et al., 2021).

There were significant differences in seropositivity by sex, age, and nationality. These are probably not due to biological differences but to differences in the likelihood of exposure to the infection. Indeed, a small proportion of the specimens tested in this study belonged to CMWs who had a higher risk of exposure to the infection than the urban population (Al-Thani et al., 2021; Jeremijenko et al., 2021). While Hamad Medical Corporation (HMC) provides healthcare primarily to the urban population and other providers cater to the CMW population, HMC is a main tertiary care center in Qatar and was also the nationally designated provider for COVID-19 healthcare needs. Thus, it is likely that a small proportion of specimens, which cannot be estimated precisely, were drawn from CMWs who were hospitalized for COVID-19 or other reasons. This may explain the higher antibody positivity of young Bangladeshi, Indian, and Nepalese men (Table 1), who form the bulk of the CMW population (Al-Thani et al., 2021; Jeremijenko et al., 2021). This may also explain the higher seroprevalence in the blood specimens drawn during inpatient or emergency clinical care, which are more likely to be COVID-19 related, than those drawn during outpatient or home care/follow-up consultation clinical care (Table 1). The higher exposure among men aged 20–69 years probably reflects their more frequent work and other activities outside the home, whereas men aged ≥70 years, urged through public health messaging to remain at home, were more likely to do so, out of concern about infection severity.

The proportion of those antibody positive who had a PCR-confirmed diagnosis prior to the antibody-positive test was 47.1% (Table 3), much higher than the 9.3% in the CMW population (Al-Thani et al., 2021), and that estimated for the total population of Qatar (11.6%) (Ayoub et al., 2021). This is probably because study specimens were drawn from individuals receiving healthcare, including those hospitalized for COVID-19, people more likely to have been tested for the infection. This fact, along with the difference in age structure between the urban and CMW populations (Planning and Statistics Authority- State of Qatar, 2020; Ministry of Interior-State of Qatar, 2020; Planning and Statistics Authority- State of Qatar, 2017; Al-Thani et al., 2021), may have resulted in higher estimates of infection severity, criticality, and fatality rates in this study (Table 3), compared to the study of the CMW population (Al-Thani et al., 2021), or model predictions for the entire population of Qatar (Seedat et al., 2020).

Strikingly, having a higher antibody titer varied by nationality, clinical care type, and time (Table 2). Variation by nationality is probably an indirect biomarker of re-exposure to infection, resulting in repeated immune system reactivation. This is suggested by the very strong positive correlation between the odds of having a higher antibody titer and seroprevalence across the nationalities (Figure 2). Lower antibody titers were found in inpatients, but this may reflect COVID-19 hospitalizations for recent infections so that there was not sufficient time for higher antibody titers to develop. There was a trend of increasing “higher antibody titers” over time, which may reflect the growing pool of infected persons who have had more time to develop higher levels of detectable antibodies after infection or alternatively to being re-exposed to the infection.

### Limitations of the study

This study has some limitations. The sample included individuals attending HMC for routine or other clinical care, but this population may not necessarily be representative of the wider urban population of Qatar. Though HMC is the main public healthcare provider and is widely accessible at minimal cost to nationals and residents, the sample may still have missed persons inclined to seek healthcare in the private sector or who avoided contact with healthcare during the COVID-19 pandemic. Some specimens may have been drawn from CMWs, who were not part of our intended study sample. However, the large sample size, equivalent to ∼10% of the urban population of Qatar, as well as the probabilistic weighting used in the analysis may have reduced inherent biases in our sample.

Laboratory methods were based on high quality, validated commercial platforms, such as the Roche platform used for serological testing (The Roche Group, 2020; Jahrsdörfer et al., 2020). The Roche platform is one of the most extensively used and investigated commercial platforms, with a specificity ≥99.8% (The Roche Group, 2020; Public Health England, 2020; Oved et al., 2020) and a sensitivity ≥89% (Jahrsdörfer et al., 2020; Abu-Raddad et al., 2021a; Oved et al., 2020). However, it is possible that the less than perfect sensitivity, especially for those with recent infections, may have underestimated the actual seroprevalence as it may take up to few weeks before recently infected individuals develop antibodies at detectable levels (Nasrallah et al., 2020; Wajnberg et al., 2020). Indeed, a recent investigation of the performance of three automated, commercial, serological platforms in Qatar, including the Roche platform, found that each of them missed ≥20% of individuals with past or current infections (Nasrallah et al., 2020). Factoring the less than perfect sensitivity and specificity (Sempos and Tian, 2021) would have increased the measured seroprevalence to 14.8% instead of 13.3%. The fatality rate may have been underestimated in this study as some individuals may have died before being tested or before developing detectable antibody levels.

In conclusion, fewer than two in every 10 individuals in the urban population of Qatar had detectable antibodies against SARS-CoV-2, suggesting that this population is still well below the herd immunity threshold and is potentially at risk from a subsequent epidemic wave. This emphasizes the need to maintain current social and physical distancing restrictions while SARS-CoV-2 vaccinations are being scaled up throughout the country. The findings also suggest that higher antibody titers appear to be a biomarker of repeated exposures to the infection.

## STAR★methods

### Key resources table

**Table:** 

REAGENT OR RESOURCE	SOURCE	IDENTIFIER
**Biological samples**		

Residual blood specimens	Hamad Medical Corporation	Not applicable

**Critical commercial assays**		

Roche Elecsys® anti-SARS-CoV-2	Roche, Switzerland	https://diagnostics.roche.com/global/en/products/params/elecsys-anti-sars-cov-2.html
TaqPath™ COVID-19 Combo Kit	Thermo Fisher Scientific, USA	https://www.thermofisher.com/order/catalog/product/A47814
AccuPower SARS-CoV-2 real-time RT-PCR Kit	Bioneer, Korea	https://us.bioneer.com/pagecat1/diagnostic/AccuPower-SARS-CoV2-RealTime-RT-PCR-Kit
cobas® SARS-CoV-2 test	Roche, Switzerland	https://diagnostics.roche.com/global/en/products/params/cobas-sars-cov-2-test.html

**Deposited data**		

Antibody testing data	This paper	Not applicable
PCR testing data	This paper	Not applicable

**Software and algorithms**		

STATA/SE 16.1	StataCorp, USA	https://www.stata.com/

### Resource availability

#### Lead contact

Further information and requests should be directed to lead contacts, Peter V. Coyle, Email: PCoyle@hamad.qa and Laith J. Abu-Raddad, Email: lja2002@qatar-med.cornell.edu.

#### Material availability

The study did not generate new reagents.

#### Data and code availability

All data are included in an aggregate form within the manuscript and its supplemental information.

### Data sources

A retrospective analysis of residual blood specimens collected from May 12 to September 9, 2020 was conducted to assess the level of and associations with antibody positivity in the urban population of Qatar. Residual blood specimens were collected from individuals receiving routine and other clinical care at Hamad Medical Corporation (HMC), a main provider of healthcare to the urban population of this country and the nationally designated provider for Coronavirus Disease 2019 (COVID-19) healthcare needs. Qatar has a universal, quality, and modern healthcare system that is heavily subsidized and equally accessible to nationals and residents. The public healthcare system is organized into several internationally accredited entities, with HMC and the Primary Health Care Corporation (PHCC) centers typically serving the urban population, and the Qatar Red Crescent Society centers typically serving the CMW population (Al-Thani et al., 2021). It follows that the tested population in this study is broadly representative of the urban population, but not of the CMW population of Qatar.

Each person in this study contributed only one antibody test, the last test performed during the study period. Antibody data generated during the study were subsequently linked to the national centralized SARS-CoV-2 polymerase chain reaction (PCR) testing and hospitalization database, which includes records for all PCR testing and COVID-19 hospitalizations in Qatar since the start of the epidemic (Hamad Medical Corporation, 2020a). The database further includes the severity classification of hospitalized cases, based on individual chart reviews completed by trained medical personnel using the World Health Organization (WHO) criteria (World Health Organization, 2020). The study was approved by the HMC and Weill Cornell Medicine-Qatar Institutional Review Boards. The study was conducted following the ethics review boards guidelines and regulations.

### Laboratory methods

Roche Elecsys Anti-SARS-CoV-2 (99.5% sensitivity (Muench et al., 2020), 99.8% specificity (The Roche Group, 2020; Muench et al., 2020); Roche, Switzerland), an electrochemiluminescence immunoassay, was used for antibody detection in the serological samples. Result interpretation followed manufacturer instructions: reactive for optical density (a proxy for antibody titer) cutoff index ≥1.0 and non-reactive for cutoff index <1.0 (The Roche Group, 2020).

Current infection was assessed using PCR testing of aliquots of Universal Transport Medium (UTM) used for nasopharyngeal and oropharyngeal swab collection (Huachenyang Technology, China). Aliquots were extracted on the QIAsymphony platform (QIAGEN, USA) and tested with real-time reverse-transcription PCR (RT-qPCR) using the TaqPath COVID-19 Combo Kit (100% sensitivity and specificity (Thermo Fisher Scientific, 2020); Thermo Fisher Scientific, USA) on an ABI 7500 FAST (Thermo Fisher, USA). Samples were also extracted using a custom protocol (Kalikiri et al., 2020) on a Hamilton Microlab STAR (Hamilton, USA) and tested using the AccuPower SARS-CoV-2 Real-Time RT-PCR Kit (100% sensitivity and specificity (Kubina and Dziedzic, 2020); Bioneer, Korea) on an ABI 7500 FAST, or loaded directly into a Roche cobas 6800 system and assayed with the cobas SARS-CoV-2 Test (95% sensitivity, 100% specificity (US Food and Drug Administration, 2020); Roche, Switzerland).

All laboratory testing was conducted at HMC Central Laboratory following standardized protocols.

### Statistical analysis

Frequency distributions were used to describe sample characteristics and optical density among antibody-positive persons. Probability weights were applied to generate estimates representing the wider urban population. Weights were developed using population distributions by sex, age group, and nationality in the PHCC database (Primary Health Care Corporation, 2020). Since the PHCC caters mainly to the urban population of Qatar through 27 geographically-distributed centers, this database, which includes 1,468,837 registered users, describes the demographics of the urban population (Primary Health Care Corporation, 2020).

Associations with anti-SARS-CoV-2 positivity, as well as with higher antibody titers (defined as optical density higher than the median value) were investigated using chi-square tests and univariable logistic regression. Covariates with p values ≤0.2 in univariable regression analysis were included in the multivariable model. Covariates with p values ≤0.05 in the multivariable analysis were regarded as strong evidence for an association with the outcome. Odds ratios, adjusted ORs, 95% confidence intervals, and p values were reported.

The antibody database was linked to the SARS-CoV-2 PCR testing and hospitalization database to enable estimation of other epidemiologic metrics. The latter included the proportion of antibody-positive persons who had a diagnosis of SARS-CoV-2 confirmed by PCR prior to the antibody test. Numbers of infections that were classified as severe, critical, or fatal, according to WHO criteria (World Health Organization, 2020), among all antibody-positive persons, were used to estimate severity, criticality, and fatality rates.

All analyses were performed in STATA/SE 16.1 (StataCorp, 2019).

## Footnotes

Supplemental information can be found online at https://doi.org/10.1016/j.isci.2021.102646.
