# Supplementary material for: SARS-CoV-2 seroprevalence in the urban population of Qatar: An analysis of antibody testing on a sample of 112,941 individuals
Source: iScience. 2021 May 24;24(6):102646. doi: 10.1016/j.isci.2021.102646 (PMC8142077; doi:10.1016/j.isci.2021.102646)
Supplement: Document S1. Figure S1 and Tables S1–S3 [file mmc1.pdf]

iScience, Volume *24*

Supplemental information

SARS-CoV-2 seroprevalence in the urban population of Qatar: An analysis of antibody testing on a sample of 112,941 individuals

Peter V. Coyle, Hiam Chemaitelly, Mohamed Ali Ben Hadj Kacem, Naema Hassan Abdulla Al Molawi, Reham Awni El Kahlout, Imtiaz Gilliani, Nourah Younes, Ghada Ali A.A. Al Anssari, Zaina Al Kanaani, Abdullatif Al Khal, Einas Al Kuwari, Adeel A. Butt, Andrew Jeremijenko, Anvar Hassan Kaleeckal, Ali Nizar Latif, Riyazuddin Mohammad Shaik, Hanan F. Abdul Rahim, Gheyath K. Nasrallah, Hadi M. Yassine, Mohamed Ghaith Al Kuwari, Hamad Eid Al Romaihi, Mohamed H. Al-Thani, Roberto Bertollini, and Laith J. Abu-Raddad

Supplemental information

Table S1. Demographic characteristics of the study sample and of the urban population of Qatar based on the Primary health Care Corporation database (Primary Health Care Corporation, 2020).

Demographic characteristics	Study sample (%)	Urban population (%)
Sex		
Women	48.4	48.0
Men	51.6	52.0
Age (years)		
<10	3.0	21.5
10-19	4.9	13.1
20-29	17.1	15.7
30-39	28.0	23.7
40-49	20.9	14.5
50-59	14.5	7.4
60-69	7.6	2.9
70-79	2.8	0.8
80+	1.2	0.3
Nationality		
All other nationalities[d]	22.0	24.8
Bangladeshi	6.9	4.7
Nepalese	4.4	1.6
Pakistani	4.5	5.6
Indian	16.5	17.8
Sri Lankan	2.0	1.6
Filipino	6.3	8.2
Sudanese	3.5	3.6
Egyptian	8.3	10.1
Qatari	25.8	21.9

Table S2. Results of regression analyses for the association with antibody positivity where antibody test date has been included as a categorical term.

Characteristics	Tested	Antibody positive		Univariable regression analysis		F test	Multivariable regression analysis	
	N (%)	N (%[a])	p-value	OR[a] (95% CI)	p-value	p-value[b]	AOR[a] (95% CI)	p-value[c]
Sex								
Women	54,707 (48.4)	4,387 (8.0)	<0.001	1.00			1.00	
Men	58,234 (51.6)	14,457 (18.3)		2.59 (2.44-2.73)	<0.001	<0.001	2.08 (1.95-2.21)	<0.001
Age (years)								
<10	3,384 (3.0)	243 (7.1)	<0.001	1.00			1.00	
10-19	5,557 (4.9)	407 (7.3)		1.04 (0.87-1.25)	0.633	<0.001	1.20 (1.00-1.45)	0.056
20-29	19,271 (17.1)	2,867 (15.0)		2.33 (2.03-2.69)	<0.001		2.03 (1.75-2.35)	<0.001
30-39	31,622 (28.0)	5,533 (16.8)		2.67 (2.33-3.06)	<0.001		2.20 (1.90-2.54)	<0.001
40-49	23,582 (20.9)	4,876 (18.1)		2.91 (2.53-3.34)	<0.001		2.45 (2.13-2.83)	<0.001
50-59	16,363 (14.5)	3,220 (17.9)		2.87 (2.50-3.31)	<0.001		2.45 (2.12-2.83)	<0.001
60-69	8,639 (7.6)	1,281 (15.0)		2.32 (2.00-2.69)	<0.001		2.10 (1.81-2.44)	<0.001
70-79	3,192 (2.8)	315 (10.5)		1.54 (1.29-1.84)	<0.001		1.71 (1.43-2.06)	<0.001
80+	1,331 (1.2)	102 (7.5)		1.06 (0.83-1.36)	0.621		1.40 (1.09-1.81)	0.009
Nationality								
All other nationalities[d]	24,799 (22.0)	2,203 (8.0)	<0.001	1.00		<0.001	1.00	
Bangladeshi	7,773 (6.9)	3,471 (41.9)		8.32 (7.66-9.04)	<0.001		5.02 (4.60-5.47)	<0.001
Nepalese	4,962 (4.4)	2,236 (38.2)		7.10 (6.43-7.85)	<0.001		4.23 (3.84-4.66)	<0.001
Pakistani	5,114 (4.5)	1,419 (23.9)		3.61 (3.23-4.04)	<0.001		3.43 (3.06-3.85)	<0.001
Indian	18,590 (16.5)	4,114 (17.5)		2.44 (2.24-2.65)	<0.001		1.95 (1.79-2.12)	<0.001
Sri Lankan	2,252 (2.0)	556 (17.3)		2.40 (2.06-2.81)	<0.001		1.84 (1.59-2.13)	<0.001
Filipino	7,085 (6.3)	1,100 (12.1)		1.59 (1.42-1.77)	<0.001		1.58 (1.41-1.78)	<0.001
Sudanese	3,954 (3.5)	466 (11.3)		1.47 (1.25-1.73)	<0.001		1.36 (1.15-1.61)	<0.001
Egyptian	9,329 (8.3)	1,150 (10.7)		1.38 (1.23-1.53)	<0.001		1.33 (1.19-1.48)	<0.001
Qatari	29,083 (25.8)	2,129 (7.1)		0.88 (0.80-0.97)	0.008		0.95 (0.86-1.04)	0.264
Clinical-care encounter type								
Emergency	18,473 (16.4)	3,333 (14.2)	<0.001	1.00		<0.001	1.00	
Inpatient	23,720 (21.0)	6,308 (19.4)		1.45 (1.35-1.57)	<0.001		1.17 (1.08-1.27)	<0.001
Outpatient	32,146 (28.5)	5,264 (13.1)		0.91 (0.85-0.98)	0.011		0.87 (0.80-0.94)	<0.001
Home care/follow-up consultations	38,602 (34.2)	3,939 (9.2)		0.61 (0.56-0.66)	<0.001		0.70 (0.65-0.76)	<0.001
Antibody test date								
16 Jun-30 Jun[e]	11,675 (10.3)	1,919 (11.9)	<0.001	1.00		<0.001	1.00	
12 May-31 May	716 (0.6)	243 (26.2)		2.62 (2.10-3.27)	<0.001		1.66 (1.34-2.07)	<0.001
01 Jun-15 Jun	2,753 (2.4)	704 (18.1)		1.63 (1.43-1.87)	<0.001		1.50 (1.31-1.73)	<0.001
01 Jul-15 Jul	16,294 (14.4)	2,555 (13.2)		1.12 (1.02-1.23)	0.021		1.21 (1.10-1.34)	<0.001
16 Jul-31 Jul	19,808 (17.5)	3,249 (13.0)		1.11 (1.01-1.21)	0.034		1.21 (1.10-1.34)	<0.001
01 Aug-15 Aug	17,319 (15.3)	3,136 (14.3)		1.23 (1.12-1.35)	<0.001		1.24 (1.13-1.37)	<0.001
16-Aug-31 Aug	29,868 (26.4)	4,759 (13.1)		1.11 (1.02-1.21)	0.018		1.35 (1.23-1.48)	<0.001
01 Sep-09 Sep	14,508 (12.8)	2,279 (13.2)		1.12 (1.01-1.24)	0.030		1.41 (1.26-1.57)	<0.001

AOR, adjusted odds ratio; CI, confidence interval; OR, odds ratio.
[a]Estimates are proportions of antibody-positive persons out of those tested weighted by sex, age, and nationality.
[b]Covariates with p-value ≤0.2 in the univariable analysis were included in the multivariable analysis.
[c]Covariates with p-value ≤0.05 in the multivariable analysis were considered strong predictors of anti-SARS-CoV-2 positivity after adjustment for sex, age, nationality, clinical-care encounter type, and antibody test date.
[d]These include other nationalities residing in Qatar.
[e]This category was chosen as a reference given the larger sample size and because of the progressive easing of the social and physical distancing restrictions since June 15, 2020 [21].

Figure S1. Trend of SARS-CoV-2 antibody positivity month-by-month. Error bars indicate 95% confidence intervals.

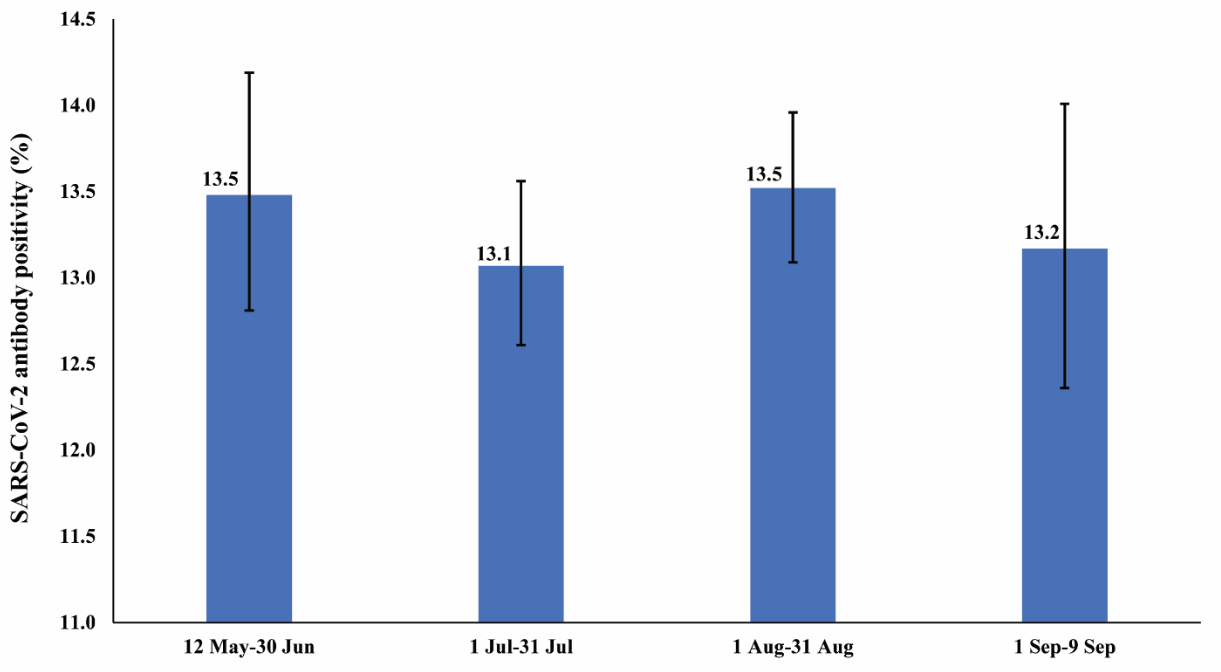

[a]Tests for the months of May and June were combined given the low number of tests in May.

Table S3. Associations for having a higher antibody titer (optical density) than the median value (≥27.0) among 18,844 antibody-positive individuals, where antibody test date has been included as a categorical term.

Characteristics	Tested	Higher antibody titers than the median		Univariable regression analysis		F test	Multivariable regression analysis	
	N (%)	N (%[a])	p-value	OR[a] (95% CI)	p-value	p-value[b]	AOR[a] (95% CI)	p-value[c]
Sex								
Women	4,387 (23.3)	2,175 (52.1)	0.702	1.00		0.702	--	--
Men	14,457 (76.7)	7,244 (51.6)		0.98 (0.88-1.09)	0.702		--	--
Age (years)								
20-29[d]	2,867 (15.2)	1,360 (47.6)	<0.001	1.00		<0.001	1.00	
<10	243 (1.3)	170 (70.0)		2.57 (1.92-3.45)	<0.001		2.94 (2.19-3.96)	<0.001
10-19	407 (2.2)	210 (54.0)		1.30 (1.02-1.65)	0.036		1.21 (0.92-1.59)	0.179
30-39	5,533 (29.4)	2,701 (48.1)		1.02 (0.92-1.13)	0.692		0.99 (0.89-1.10)	0.816
40-49	4,876 (25.9)	2,442 (50.1)		1.11 (1.00-1.22)	0.051		1.16 (1.04-1.30)	0.007
50-59	3,220 (17.1)	1,634 (50.8)		1.14 (1.02-1.27)	0.020		1.24 (1.10-1.40)	<0.001
60-69	1,281 (6.8)	700 (54.2)		1.31 (1.14-1.50)	<0.001		1.51 (1.30-1.76)	<0.001
70-79	315 (1.7)	158 (49.7)		1.09 (0.86-1.38)	0.480		1.43 (1.11-1.84)	0.005
80+	102 (0.5)	44 (41.7)		0.79 (0.52-1.19)	0.254		1.30 (0.83-2.02)	0.247
Nationality								
All other nationalities[e]	2,203 (11.7)	1,064 (51.7)	<0.001	1.00		<0.001	1.00	
Bangladeshi	3471 (18.4)	1,946 (56.2)		1.20 (1.04-1.38)	0.012		1.69 (1.46-1.95)	<0.001
Nepalese	2,236 (11.9)	1,099 (49.3)		0.91 (0.78-1.06)	0.210		1.54 (1.32-1.81)	<0.001
Filipino	1,100 (5.8)	507 (46.2)		0.80 (0.65-0.99)	0.038		1.31 (1.05-1.62)	0.016
Indian	4,114 (21.8)	2,027 (51.0)		0.97 (0.84-1.13)	0.699		1.23 (1.05-1.43)	0.010
Pakistani	1,419 (7.5)	731 (55.5)		1.16 (0.96-1.41)	0.124		1.19 (0.98-1.44)	0.077
Sri Lankan	556 (3.0)	242 (45.2)		0.77 (0.59-0.99)	0.044		1.11 (0.87-1.43)	0.395
Sudanese	466 (2.5)	264 (53.2)		1.06 (0.78-1.44)	0.712		1.04 (0.77-1.40)	0.802
Egyptian	1,150 (6.1)	535 (49.0)		0.90 (0.73-1.10)	0.283		0.83 (0.68-1.01)	0.068
Qatari	2,129 (11.3)	1,004 (51.8)		1.00 (0.84-1.21)	0.959		0.80 (0.66-0.96)	0.018
Clinical-care encounter type								
Emergency	3,333 (17.7)	1,866 (58.5)	<0.001	1.00		<0.001	1.00	
Outpatient	5,264 (27.9)	3,189 (60.7)		1.10 (0.97-1.25)	0.136		1.11 (0.98-1.26)	0.113
Home care/follow-up consultations	3,939 (20.9)	2,195 (58.3)		0.99 (0.86-1.14)	0.915		1.03 (0.90-1.19)	0.628
Inpatient	6,308 (33.5)	2,169 (35.2)		0.39 (0.34-0.44)	<0.001		0.39 (0.34-0.45)	<0.001
Antibody test date								
12 May-30 Jun	2,866 (15.2)	846 (31.1)	<0.001	1.00		<0.001	1.00	
01 Jul-15 Jul	2,555 (13.6)	1,190 (46.2)		1.91 (1.62-2.24)	<0.001		1.83 (1.53-2.18)	<0.001
16 Jul-31 Jul	3,249 (17.2)	1,649 (52.4)		2.44 (2.09-2.85)	<0.001		2.22 (1.88-2.63)	<0.001
01 Aug-15 Aug	3,136 (16.6)	1,745 (56.0)		2.82 (2.41-3.29)	<0.001		2.51 (2.13-2.96)	<0.001

16-Aug-31 Aug	4,759 (25.3)	2,719 (58.6)	3.14 (2.72-3.62)	<0.001		2.68 (2.29-3.13)	<0.001
01 Sep-09 Sep	2,279 (12.1)	1,270 ((58.3)	3.10 (2.60-3.68)	<0.001		2.52 (2.10-3.03)	<0.001

AOR, adjusted odds ratio; CI, confidence interval; OR, odds ratio.

[a]Estimates are proportions of persons with antibody titers higher than the median among those antibody-positive, weighted by sex, age, and nationality.

[b]Covariates with p-value ≤0.2 in the univariable analysis were included in the multivariable analysis.

[c]Covariates with p-value ≤0.05 in the multivariable analysis were considered strong predictors of anti-SARS-CoV-2 positivity after adjustment for age, nationality, clinical-care encounter type, and antibody test date.

[d]The 20-29 age group was chosen as the reference group (instead of the <10 age group) because of the larger sample size and for epidemiological relevance.

[e]These include other nationalities residing in Qatar.
